# Unpacking the early implementation of social prescribing in Swedish primary care: a theory-informed process evaluation

**DOI:** 10.1093/heapro/daag041

**Published:** 2026-03-20

**Authors:** Frida Jonsson, Emilia W E Viklund, Frida Degerstedt, Anna Sofia Lundgren, Ingeborg Nilsson

**Affiliations:** Department of Social Work, Samhällsvetarhuset, plan 5, Biblioteksgränd 4, Umeå University, 901 87 Umeå, Sweden; Department of Community Medicine and Rehabilitation, Biologihuset, plan 6, Johan Bures väg 12, Umeå University, 901 87 Umeå, Sweden; Department of Community Medicine and Rehabilitation, Biologihuset, plan 6, Johan Bures väg 12, Umeå University, 901 87 Umeå, Sweden; Department of Culture and Media Studies, Humanisthuset, Biblioteksgränd 3, Umeå University, 901 87 Umeå and Affiliated with Centre for Demography and Ageing Research (Cedar), Norra Beteendevetarhuset, plan 4, Humanioragränd, Umeå University, 901 87 Umeå, Sweden; Department of Community Medicine and Rehabilitation, Biologihuset, plan 6, Johan Bures väg 12, Umeå University, 901 87 Umeå, Sweden

**Keywords:** social prescribing, primary care, loneliness, older adults, implementation, process evaluation, Normalization Process Theory (NPT)

## Abstract

Social prescribing has gained attention internationally as a health-promoting way to address patients’ nonmedical needs, yet research in the Nordic healthcare context remains limited. This theory-informed process evaluation set out to unpack the early implementation of a social prescribing model in Swedish primary care, developed to reduce loneliness and promote health among older adults. Guided by Normalization Process Theory (NPT) and the Consolidated Framework for Implementation Research (CFIR), this study assessed how and under what conditions the model was implemented across 10 primary care centres between May 2023 and December 2024. Data were triangulated from interviews with prescribers and patients, alongside prescribing and follow-up materials. Thematic analysis indicated that while the approach was perceived as relevant and timely, implementation was hindered by a selective use of the routine screening question and lack of person-centredness in the prescribing process, reflecting poor fidelity to core principles. Leadership engagement and managerial support that appeared symbolic rather than active further constrained implementation by limiting integration into organizational workflows, rendering the approach peripheral or optional to other clinical and statutory tasks. The model’s operationalization also lacked alignment with community resources and bridging structures, limiting implementation by failing to meet patients’ needs and expectations. While the Swedish social prescribing approach aligns with national policy discourse and prescribers’ values, its scale-up will require clearer guidance on components needing high-fidelity delivery, necessary organizational structures, accessible local resources, and mechanisms linking primary care with the wider community. These findings contribute to the international evidence base on social prescribing and inform refinements of the Swedish model.

Contribution to Health Promotion

- Social prescribing is a way for primary care to shift from traditional disease-centred and curative approaches towards proactive models of care.
- By addressing loneliness, social prescribing can complement clinical services and meet nonmedical needs of older adults.
- Implementation insights show that social prescribing in Sweden aligns with national health system reforms emphasizing health promotion and person-centredness.
- Preconceived ideas about who is lonely or what it looks like hindered implementation through selective screening for loneliness among older adults.
- Weak organizational support and poor links to local resources constrained integration into routine care and community networks.

## Introduction

Demographic and epidemiological changes, alongside workforce shortages and financial pressures, have intensified challenges for health systems in many high-income countries during the last decades (Polin *et al*. 2021). To manage these developments in caring for aging populations with chronic conditions, complex comorbidities, and multifaceted needs, traditional disease-centred and curative approaches have been considered increasingly inadequate (WHO 2018). Hence, it is recognized that health systems, especially the frontline services of primary care, must shift from isolated, medicalized interventions towards more proactive, integrated, and person-centred models of care (Valentijn *et al*. 2013). One example of such models is social prescribing (WHO 2022, Bertotti 2024). Although definitions vary, this approach has developed to address patients’ broader needs by connecting them with nonclinical resources in the local community (Muhl *et al*. 2023). This means that social prescribing can be tailored to specific groups, but it is also generally applicable by offering support with a range of social, emotional, and practical issues (Oster *et al*. 2023, Scarpetti *et al*. 2024).

The UK has led the way in developing and implementing social prescribing, although the evidence remains weak and inconclusive regarding patient outcomes and value for money (Bickerdike *et al*. 2017, Chatterjee *et al*. 2018, Pescheny *et al*. 2020, Costa *et al*. 2021, Vidovic *et al*. 2021, Kiely *et al*. 2022, Napierala *et al*. 2022). Despite this, the approach has gained momentum internationally, with initiatives ranging from pilot projects to national rollouts across Europe, Asia, Australia, and North America, although research has been limited in the Nordic context (Morse *et al*. 2022, Scarpetti *et al*. 2024). This means that the use of social prescribing spans diverse geographies and health systems, evolving more rapidly than its evidence on effects and cost-effectiveness (Alderwick *et al*. 2018, Savage *et al*. 2020, Kiely *et al*. 2022, Percival *et al*. 2022). In the current study, we contribute to the evidence by sharing insights from Sweden, where a social prescribing model has been developed for and tested in primary care as part of a research project (Johansson *et al*. 2021). However, rather than assessing its impact, we focus on the processes through which the model was implemented to reduce loneliness and improve health among older adults.

This study aims to unpack the early implementation of social prescribing—a new and complex intervention—within the dynamic and ‘real-world’ context of Swedish primary care (Moore *et al*. 2015). Through a theory-informed process evaluation, we integrate Normalization Process Theory (NPT) with the Consolidated Framework for Implementation Research (CFIR), to assess how and under what conditions the model was implemented between May 2023 and December 2024 at 10 primary care centres (Skivington *et al*. 2021, Schroeder *et al*. 2022). In addition to generating evidence on the procedures and practices of social prescribing internationally, this study provides a basis for further refinement of the Swedish model by offering insights into its feasibility and potential for scale-up in primary care (Johansson *et al*. 2021).

### Delivery and implementation of social prescribing

**Figure 1:**
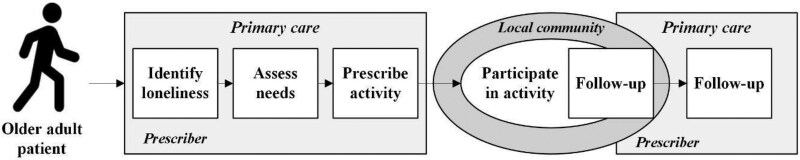
Steps of the Swedish social prescribing model (in squares).

Like other examples (Morse *et al*. 2022, Oster *et al*. 2023, Scarpetti *et al*. 2024), the Swedish social prescribing model was situated in primary care. While this was not mandated by policy but a deliberate choice of the research project (Johansson *et al*. 2021), it followed national developments seeking to reorient the health system toward more proactive and health-promoting approaches (SOU 2018:39) in line with global trends (WHO 2018). In addition, consistent with awareness of loneliness as a pressing public health problem internationally (WHO 2025) and in Sweden (Swedish Public Health Agency 2025), the model was directed toward lonely older adults rather than a general population of anyone with nonmedical needs (Oster *et al*. 2023). Following social prescribing schemes that include more intensive and extended forms of support (Polley *et al*. 2017, Husk *et al*. 2020, Tierney *et al*. 2020), the Swedish model comprises a four-step, person-centred process (Fig. 1).

Below, we outline the steps of the model, with references to international literature being made for context rather than systematic comparison.

#### Identify loneliness

In some countries, healthcare staff are reassigned to deliver social prescribing, while in others, new roles, such as ‘well-being coordinators’ and ‘community connectors’, are created, where the English ‘link worker’ is the most established (Morse *et al*. 2022). In Sweden, the model was developed to use available resources within primary care, aiming to integrate the approach into ‘real-world’ conditions and avoid formalizing new functions alongside existing ones. This meant that primary care providers had discretion to decide which licenced professionals would act as ‘social prescribers’, resulting in nurses, psychologists, and particularly occupational therapists assuming the role as part of their clinical responsibilities.

As a first step of the model, these prescribers are expected to identify lonely older adults during regular primary care encounters using a ‘standardized screening tool’ in the form of a routine question (‘are you troubled by loneliness?’). Patients who respond affirmatively, regardless of their reason for seeking care, are then provided information about social prescribing and offered to try the approach. Those who express a need or interest are then referred to a second meeting with the prescriber for a more comprehensive assessment.

#### In-depth assessment

After being referred a patient who expresses being troubled by loneliness as well as a need for and interest in the social prescribing approach, the prescriber conducts a comprehensive, yet ‘in-depth assessment’ to explore interests in, needs for, and abilities to engage with social activities. This contrasts with other international models that are either focused on more specific activities (e.g. exercise or art) or adopt a broader focus on general nonmedical needs (Oster *et al*. 2023). However, in line with notions of ‘matching’ (Tierney *et al*. 2022 ), the Swedish model includes an explorative checklist to support prescribers in identifying patients’ preferences and prerequisites for social activities while building on their knowledge of options available in the local community.

#### Personalized prescription

As a more specific form of referral and action planning, the Swedish assessment is then translated into a ‘personalized prescription’ that outlines the activity to participate in, including details on when, where, and for how long, as well as the goal of participation. This is both in line with and distinct from other social prescribing schemes, where the process can be less formalized, often relying on signposting and verbal suggestions, but also highly structured, following a tangible and coproduced plan (Husk *et al*. 2020, Tierney *et al*. 2020). Consistent with the literature, the Swedish model’s emphasis on a written prescription that articulates personal goals and actionable steps draws on principles of collaborative planning and motivational support. This formalized approach may be particularly important for older adults, who often benefit from clarity and structure to enhance engagement and follow-through (Percival *et al*. 2022).

#### Repeated follow-ups

The final step sets a structure for ongoing support, reflecting what Husk *et al*. (2020) have described as a ‘connected approach’. This specifies the need for prescribers to sustain patients’ engagement by conducting ‘repeated follow-ups’ at least two times: one by phone after about 3 weeks to check progress and make any necessary adjustments to the prescription and another in person around 3 months later to assess experiences and potential impacts of attending the activity. However, unlike other international examples, where patients receive sustained support, such as ‘buddying’ from a link worker in transitioning to and engaging with the activity (White *et al*. 2022), the final step of the Swedish model is more directive. While this requires less work for the prescriber in primary care, it places greater responsibility on the patient to independently manage the transition from prescription to participation in a community-based social activity.

### Framework integrating Normalization Process Theory and Consolidated Framework for Implementation Research

From an implementation perspective, the Swedish model defines its core principles through four steps as detailed in Fig. 1: identification through screening, in-depth needs assessment, personalized prescriptions, and structured follow-ups. By comprising these steps, which include various interacting elements shaped by factors operating at different levels, the model is best understood as a complex intervention (Skivington *et al*. 2021). To account for this complexity in assessing how and under what conditions the model was implemented, we follow Schroeder *et al*. (2022) by integrating NPT (May and Finch 2009) and CFIR (Damschroder *et al*. 2022) as a combined theoretical framework.

The combination of theories allows us to capture both processes and contextual conditions, enabling a more comprehensive analysis than either one could offer alone. While NPT guides us to examine how prescribers and patients interpreted and understood the approach, CFIR provides a lens to examine the conditions under which these processes unfolded. The need for such a combined perspective builds on the idea that complex interventions, such as social prescribing, are not fixed sets of externally defined components but ‘material practices’, comprising ensembles of beliefs, behaviours, and relationships intended to (re)organize people, objects, and systems (May and Finch 2009). In this sense, implementation is neither a technical exercise nor about automated transfer of knowledge, but about how ‘people’ enact the intervention and embed it into context through everyday understandings, decisions, and (inter)actions. According to NPT and supported by CFIR (May and Finch 2009, Damschroder *et al*. 2022), this occurs via four mechanisms that vary in importance across settings:

1. ‘Coherence’—how individuals understand and make sense of the intervention, both on its own and in relation to other practices. By being contingent on the meanings that are created and internalized, this shapes implementation by anchoring it in knowledge and experiences.
2. ‘Cognitive participation’—who gets involved, how, and why. Building on its legitimacy and perceived relevance, this shapes implementation by securing commitment and ‘buy-in’ of actors—i.e. the emotional and social investments that make the intervention worth doing.
3. ‘Collective action’—how the intervention is operationalized. This shapes implementation as people coordinate and collaborate by negotiating roles, responsibilities, and resources.
4. ‘Reflexive monitoring’—how the intervention is appraised and adapted over time. This helps to sustain or reshape implementation based on perceived effects, relevance, and value.

Together, these mechanisms offer a framework for analysing the dynamics of implementing social prescribing in real-world settings. However, while NPT focuses on how implementation ‘gets done,’ it also acknowledges that this does not occur in a vacuum but is rooted in, and often constrained by, various contextual conditions (May and Finch 2009, May *et al*. 2016). CFIR provides complementary perspectives on these influences (Damschroder *et al*. 2022, Schroeder *et al*. 2022). In an ‘outer setting’, comprising the wider context external to the intervention, factors such as policy pressures, public expectations, and community needs can shape implementation by affecting motivations, knowledge, and behaviours of both implementers and recipients. For community-based interventions like social prescribing, implementation may also depend on the availability of local resources and the existence of networks, partnerships, or infrastructures that connect these resources to actors and processes within the ‘inner setting’ (Damschroder *et al*. 2022).

The inner setting, comprising the context more immediate to the intervention, acts not merely as a backdrop to, but as an active force in, implementation (Dopson and Fitzgerald 2005). Leadership commitment and managerial support are crucial as they help set internal priorities, secure resources, allocate time, and facilitate communication. These supports enable implementers to embed the intervention into routines and workflows, either by adapting it to fit the context or by reconfiguring the context to accommodate the intervention. This balancing act reflects what May *et al*. (2016) have described as ‘intervention plasticity’ and ‘contextual elasticity’. Plasticity refers to the capacity of an intervention to be tailored to local conditions without compromising core functions that should be implemented with high fidelity, while elasticity denotes the extent to which the context can be stretched or reshaped to support the intervention, whether it is adapted or not (May *et al*. 2016).

In sum, the above conditions illustrate the dynamic interplay between intervention and context, underscoring the importance of both adaptability and alignment for successful implementation.

## Materials and methods

### Study setting and design

This study is part of the research project ‘Social Prescribing in Sweden (SPiS)’, which has pioneered the development and early implementation of a social prescribing model tailored to Swedish primary care aiming to reduce loneliness and promote health among older adults (Johansson *et al*. 2021). Consistent with the Medical Research Council (MRC) framework for developing and evaluating complex interventions (Skivington *et al*. 2021), the model was codeveloped by the research team with professionals from primary care and actors in one municipality. The development process, starting in 2018, was iterative and participatory, structured around workshops where international evidence and local experiences were presented, discussed, and adapted the Swedish context. This resulted in the gradual formation of a step-by-step model, as detailed above, which was first tested and successively refined at one primary care centre, located in a northern Swedish municipality before being formalized through written and visual materials.

As part of the project, the research team moved into an extended feasibility phase in 2023, where the model was further tested across 10 additional primary care centres located in six municipalities (five centres were in the same municipality and collaborated during the project) until the end of 2024. The aim of this feasibility testing was to evaluate both the early implementation and effectiveness of the model in real-world settings (Skivington *et al*. 2021). Specifically, while the project comprised a pragmatic randomized controlled feasibility trial (Degerstedt *et al*., submitted for publication), including both quantitative and qualitative components, the present study report results from analyses of qualitative data. To facilitate this, a process evaluation was conducted (Moore *et al*. 2015), integrating NPT and CFIR as theoretical frameworks (Schroeder *et al*. 2022).

### Recruitment of primary care centres

Primary care centres were recruited through self-initiative after outreach via news articles, presentations, and other media. Centres were eligible if they (i) viewed loneliness among older adults as a pressing issue, (ii) had managerial and prescriber support, and (iii) were willing to test and help develop the model. Interested centres engaged in:

- Digital introductory meetings on the model and research setup
- On-site meetings to review procedures in detail
- Follow-ups every 2–3 months during implementation to address progress and challenges

Centres also received access to a webpage with model details, case examples, resources on loneliness, and research team contacts. No interested and eligible centres were excluded, although one withdrew after preparatory activities. Ten centres thereby continued implementation, representing a mix of rural and urban settings and both public and private providers (Table 1).

**Table 1:** Overview of participating primary care centres.

	Location	Setting	Provider type	Prescribers
Centre A	North	Rural	Public	One occupational therapist One healthcare counsellor
Centre B	Central	Rural	Public	One occupational therapist One psychologist
Centre C	Central	Rural	Public	One assistant nurse One rehabilitation coordinator
Centre D	South	Urban	Private	Two occupational therapists
Centre E	South	Urban	Private	One nurse
Centre F	Central	Urban	Public	Four occupational therapists
Centre G				
Centre H				
Centre I				
Centre J				

### Data collection

Consistent with the process evaluation design (Moore *et al*. 2015), this study triangulates qualitative data collected through interviews with prescribers conducted during and after implementation. It also includes interviews with older adult patients who were recruited to the project and had experience with the approach as well as information from prescribing materials, such as prescriptions and prescriber notes from the follow-ups. The interviews were conducted by four authors (F.J., E.W.E.V., F.D., and I.N.), all experienced qualitative researchers and familiar with the model. Guides tailored for the different types of interviews and participant groups were used to ensure consistency across interviews, while the semistructured format allowed flexibility to probe emerging issues, clarify meanings, and adapt to participants’ experiences. These guides were developed collaboratively and iteratively refined based on the study aim, theoretical framework, and emerging insights, with questions organized thematically as detailed below.

#### Prescriber interviews

As a first step, and in parallel with the implementation (May 2023–December 2024), we conducted 4–5 short, semistructured longitudinal interviews with prescribers at each centre (27 in total). These interviews were conducted digitally every 2–3 months by one or two of the interviewing authors, lasting ∼20–30 minutes. Hence, while the majority were group interviews, they were conducted individually with prescriber at Centre E since she was working alone with the approach. While the interviews were audio recorded with consent, data was collected as analytical notes rather than verbatim transcripts. These interviews focused broadly on how the work was progressing, including three open-ended questions on (i) challenges, (ii) opportunities, and (iii) ideas on the next steps. However, the interviews also had a formative function by enabling discussions of ongoing issues in ways that could support the implementation process.

Informed by preliminary analyses of notes from the longitudinal interviews, three authors (E.W.E.V., F.D., and I.N.) individually visited the centres between March and June 2025 to conduct semistructured, postimplementation interviews with prescribers. These interviews (six in total, since centres in the same municipalities attended the same interview) were conducted face-to-face, guided by open-ended questions on conditions around and experiences of (i) asking about loneliness and recruiting patients, (ii) assessing needs for and prescribing social activities, (iii) supporting participation in prescribed activity, and (iv) any other issues or reflections. Four of these interviews were in groups, while two were individual with prescribers at Centres E (nurse) and B (occupational therapist).

#### Patient interviews

While visiting the centres to conduct postimplementation interviews, E.W.E.V., F.D., and I.N. also conducted individual semistructured interviews with patients who had participated in the project. These patients had been successively recruited by prescribers during the implementation based on the criteria: (1) being aged 65 or older, (2) being bothered by loneliness, (3) being interested in trying social prescribing, and (4) consenting to participate in the research and for contact details and other social prescribing material to be shared with the research team. All patients who consented to participate in the project were contacted in spring 2025 and invited to an interview, conducted either face-to-face or by phone according to their preference. Of these, 16 agreed to participate, 11 women and 5 men aged 69–92 years. Besides capturing general perceptions of the model and support received, the patient interviews explored experiences of (i) being identified and referred as well as of participating in the (ii) needs assessment, (iii) prescription process, and (iv) prescribed activities. While most interviews were digitally recorded with consent and transcribed verbatim, a few patients declined recording, and data were then documented as field notes.

#### Prescribing material

Data were also drawn from written prescriptions and prescriber notes from the follow-ups after 3 weeks and 3 months. Structured templates supported these follow-ups, including guiding questions and space for notes. The 3-week template focused on adherence and potential revisions; the 3-month template asked whether the prescription was followed, suited the patient, and required changes, with space for explanations. For this study, we analysed 18 prescriptions, 16 three-week templates, and 7 three-month templates alongside interviews.

### Data analysis

Data from the interviews and prescribing material was analysed using an abductive approach to reflexive thematic analysis (Braun and Clarke 2021). The analysis began with repeated listening to audio recordings and rereading of analytical notes from the longitudinal interviews. This initial phase, led by the first author in close dialogue with the rest of the team, involved memoing, followed by inductive coding and categorization to identify recurring patterns and emerging themes related to the implementation process across centres. Preliminary findings informed the subsequent postimplementation interviews with prescribers and patients. Transcripts from these interviews were then analysed following an iterative process coding, categorization, and thematization.

The first author started with an abductive coding of the prescriber interviews. Descriptive and interpretive codes were generated based on close engagement with the data and informed by NPT and CFIR, being subsequently integrated and abstracted into preliminary themes that captured higher-order patterns of meaning. The same process was then followed for the patient interviews and the prescriber material, with preliminary findings being discussed among the authors. Themes were then iteratively refined by synthesizing across centres and sources of data, integrating data-driven insights with theory-informed interpretations. This approach supported a nuanced understanding of early implementation challenges and opportunities across diverse settings (May and Finch 2009).

### Ethical approval

The study was approved by the Swedish Ethical Review Authority (Dnr 2020-00659) and has been conducted in accordance with the World Medical Association Declaration of Helsinki. Participation was voluntary, and written informed consent was obtained from all participants after they had been informed about the study’s purpose, procedures, potential implications, and their right to withdraw. All data were handled in compliance with applicable ethical and legal standards, ensuring secure storage, sharing, and processing of both personal data and sensitive personal information.

While the study integrates two complementary frameworks, adopts a longitudinal qualitative design, and triangulates data across 10 primary care centres, it also has limitations. First, sampling focused mainly on patients and prescribers, as few other staff members had direct experience with the model, limiting insights into broader organizational perspectives. Second, since participation was voluntary and largely based on interest, we have likely attracted centres, prescribers, and patients more positive towards new approaches. However, aiming for transferability rather than generalizability, the results may offer insights relevant to similar contexts (Drisko 2025). Third, although prescribers discussed challenges, they may have presented their experiences in a more positive light due to perceived expectations from the research team or colleagues, particularly in group interviews.

## Results

The findings from this process evaluation primarily reveal difficulties in implementing the Swedish social prescribing approach across 10 primary care centres. As detailed below, the results highlight several issues that negatively shaped how the model was enacted and embedded, both in the more immediate primary care setting and related to the wider community context. As such, while some positive aspects were seen, with the approach appearing to have been (a) ‘relevant initially or in theory’, implementation was largely constrained because, in reality, it was (b) ‘not consistent with perceptions of loneliness’, (c) ‘not compatible with perceived organizational practice’, and (d) ‘not meeting patients’ needs and expectations’. In the following, we present these findings under four thematic headings, integrating aspects of our theoretical framework.

### Relevant initially or in theory, but…

According to NPT, alignment between a new intervention and existing policies, perceived problems, and professional identities can support implementation by fostering coherence (how people make sense of the intervention) and cognitive participation (how and why they engage with it) (May and Finch 2009). Similarly, CFIR highlights the importance of both outer and inner setting factors, such as policy and professional fit, in enabling implementation (Damschroder *et al*. 2022). When an intervention is consistent with policy discourse, addresses a recognized and relevant challenge, and resonates with practitioners’ values and expertise, it is more likely to be perceived as legitimate, worthwhile, and actionable, thus increasing engagement and motivation to implement.

The Swedish social prescribing model appears to tick all these boxes. It aligns with national policies aiming to reorient the health system towards more proactive, person-centred, and health-promoting approaches (SOU 2018:39) while responding to a problem—loneliness among older adults—that has received growing public attention, particularly in the wake of COVID-19 (Swedish Public Health Agency 2025). It was partly against this background that the primary care centres voluntarily joined the project, with managers and prescribers expressing explicit concerns about loneliness as a pressing and underaddressed issue in primary care. Many described encountering loneliness in clinical practice prior to participating in the project yet lacked tools to address it through conventional medical interventions. The social prescribing model was therefore perceived as both timely and relevant, presenting a novel approach to addressing a well-recognized gap in care:

/…/that it’s our responsibility too. To see the patient. We see the problem if you are lonely [and] when we see the problem, being able to solve it, not just… just give you medication. (prescriber, Centre E)

Since most prescribers were occupational therapists, the approach also largely resonated with their professional identities and competencies: they often already worked holistically, considering social and emotional dimensions of health, while being trained to support engagement in activities. This alignment supported coherence and cognitive participation (May and Finch 2009), as prescribers felt social prescribing was both appropriate to and relevant within their scope of practice. Rather than requiring a radical reorientation of roles, the model was viewed as compatible with their responsibilities and as a complement to the daily work, which helped secure early engagement and buy-in (May and Finch 2009). However, despite this alignment, which appeared to have spurred initial motivation, the implementation proved challenging. One prescriber at Centre A described that they were ‘really excited *for a while* [our emphasis]’, highlighting how early enthusiasm, for some, did not necessarily translate into sustained engagement. However, for others, engagement appears to have been more consistent, despite the implementation being hampered by a range of factors as detailed below.

### … not consistent with perceptions of loneliness

Perceiving a new intervention as relevant by responding to a pressing problem is recognized as a key aspect of enabling implementation (May and Finch 2009, Damschroder *et al*. 2022). However, as our findings suggest, it is not only the perceived relevance of the problem that matters, but also how the problem itself is understood, and how the intervention is interpreted as a potential or proper response. In this regard, our analysis indicates that prescribers’ understandings and perceptions of loneliness significantly influenced the enactment of a core component of the Swedish model: routinely asking older adult patients, ‘Are you troubled by loneliness?’.

Although a central aspect of the model was to integrate the standardized screening question into all clinical encounters with older adults, our analysis suggests that it was not consistently applied within and across settings. While some prescribers appeared comfortable with the topic and did not perceive the question as problematic, others expressed uncertainty or discomfort, both about loneliness and the appropriateness of asking about it directly, as illustrated in the following quote:

/…/if it is a memory assessment, then it is easier to bring up the question of loneliness than if you have a patient with hand problems. In that case, it does not feel as natural to ask about it. So, it depends on the person you have in front of you. (prescriber, Centre A)

Some prescribers described reformulating the question to make it less confrontational, such as asking about social contacts rather than experiences of loneliness, indicating a need to adapt the intervention to fit their communication style and perceived patient preferences. Others discussed the challenges of introducing the topic in time-constrained and medicalized contexts of primary care, where loneliness might not seem immediately relevant, be considered low priority, or fall outside the scope of clinical responsibilities. However, regardless of whether the topic itself or the act of asking was perceived as problematic, most prescribers appeared to use the question selectively despite our efforts to remind them about asking (such as with pins and folders). As discussed elsewhere, prescribers’ discretion in asking appeared to be largely shaped by their understandings of loneliness. In terms of implementation, this translated into patients being asked primarily if they ‘appeared’ or could ‘potentially be’ lonely, based on prescribers’ judgements and preconceived ideas about who is lonely or what loneliness looks like. One prescriber expressed it as follows: ‘It’s when you suspect that this [troubled by loneliness] might be the case that you ask the question’. Such understandings seemed to also contribute to the question being avoided in situations where the prescriber felt unprepared to manage the response, where it felt awkward to bring it up, or where it was perceived as potentially offensive or intrusive.

The varied perceptions of loneliness meant that the intervention’s reach was uneven, but also insufficient, as prescribers (un)intentionally decided who might be lonely and thus potentially eligible for, and in need of, the intervention. This selective enactment constrained implementation to a considerable degree. For example, while each primary care centre was expected to recruit 20 patients, and prescribers initially expressed confidence in reaching this target, only 27 patients were recruited ‘in total’ after 10–18 months of implementation across the 10 centres, far below the expected 200. Further, in line with the idea that implementation is not just about doing, but about sense-making (May and Finch 2009), prescribers’ perceptions of loneliness appeared to shape how the model was understood as a response to address it. Rather than being an approach that should be equitably provided based on need, in line with Swedish healthcare policy (SFS 2017:30), it indirectly became a targeted solution, potentially reaching only a specific subset of patients who could ‘fit’ a predetermined profile of loneliness or was perceived as able to engage with social activities. For example, most prescribers seemed to think that older adults with cognitive difficulties were not a suitable target group, even if they might experience loneliness.

The selective approach emerging from preconceived understandings of loneliness contrasts with established definitions, which considers it a subjective experience that arises when one’s social life does not meet one’s social needs (Prohaska *et al*. 2020). The selection may also have limited the intervention’s potential to normalize stereotypical conventions about loneliness and broaden access to supportive activities. In this way, the discretionary practices used to operationalize the model (i.e. selectively asking the question) may have inadvertently narrowed its scope and implementation via gatekeeping processes. This, in turn, might undermine the model’s broader aim of reducing loneliness among older adults, but it may also introduce or increase health inequities (Lorenc *et al*. 2013).

### … not compatible with perceived organizational practice

Besides being shaped by prescribers’ perceptions of loneliness and the selective application of the core questions, implementation was significantly influenced by factors in the immediate primary care context. According to CFIR, leadership commitment and support within the inner setting is often necessary, but not always sufficient, for implementation (Damschroder *et al*. 2022). Equally important is the intervention’s compatibility with existing workflows and routines, as well as its relative advantage over ordinary practices—i.e. if it adds value or simply competes with, or distracts from, regular and statutory tasks (May and Finch 2009, Damschroder *et al*. 2022).

While local leadership commitment was an inclusion criterion for primary care centres to join the project, this support appeared symbolic and passive rather than actively encouraging or championing the approach. In practice, managerial engagement was inconsistent. At one centre, prescribers described feeling supported and were able to prioritize the work, facilitating implementation. In contrast, prescribers at another centre noted that although participation in the project was formally sanctioned, they had no ‘clear mission’ or mandate to work with the approach. This left them to navigate its operationalization at their own discretion, without apparent directions and organizational legitimacy. The perceived lack of managerial support meant that prescribers also had to advocate for the model themselves and take responsibility for engaging others in asking the routine question, which was perceived as challenging in a context shaped by time constraints, high staff turnover, and competing priorities. One prescriber explained that although there were at least 10 general practitioners on site, ‘there were very few who seemed to embrace the approach’. At a third centre, the prescriber described how conflicting demands negatively shaped managerial attitudes:

The manager was… It’s because there was a lot of COVID vaccines and we had few nurses working on it/…/. So, there was a lack of time. So, that’s why he was a little against it at first. But when it didn't have to take time from ordinary practices or cost money, then it worked. (prescriber, Centre E)

As illustrated in the quote and echoed by prescribers at another centre who stated that ‘we have an order of prioritisation, and other things take priority,’ the intervention appeared to conflict with more urgent clinical and statutory responsibilities. The symbolic leadership support also meant that prescribers had to manage internal priority setting themselves, with tasks being added to rather than removed from an already hectic schedule when social prescribing was introduced. From a CFIR perspective (Damschroder *et al*. 2022), this highlight how the model struggled to demonstrate an advantage and was instead seen as competing with ordinary practices. Through the lens of NPT (May and Finch 2009), the lack of managerial engagement seemed to further weaken the collective action needed to embed the approach into workflows and routines, making it a side activity, peripheral or optional to core tasks. As such, the model’s misalignment with perceived organizational practice, compounded by limited managerial engagement, undermined its implementation and potential to become a sustained part of the primary care centres.

### … not meeting patients’ needs or expectations

Successful implementation depends on how practitioners make sense of an intervention and engage with it, but also on how it is operationalized, what NPT refers to as ‘collective action’ (May and Finch 2009). Similarly, CFIR highlights various outer setting aspects, bridging factors that link outer and inner settings, and patient characteristics, which influence how an intervention fits with local contexts and corresponds to individual needs (Damschroder *et al*. 2022).

While a core aspect of the Swedish model was to offer personalized support to help lonely older adults identify and engage in meaningful social activities, our findings suggest that the ways in which the model was operationalized did not always align with their needs, preferences, or expectations. In some cases, the identified activities did not seem to reflect what the patient needed or wanted, revealing a lack of fidelity to person-centredness in the prescribing process. Activities appeared to often be prescribed based on what was administratively feasible or available in the local community, rather than what was meaningful to the individual. Many patients appeared to need activities that offered emotional support (e.g. coping with the loss of a spouse) or opportunities for companionship (e.g. finding a friend or partner), rather than being referred to structured or general social group activities. Results from both interviews and prescribing material indicated that the most prescribed activities were general ones held at common meeting places (e.g. churches and senior centres) or related to physical activity, which appeared to lack the relational depth that many older adults were seeking. Prescribers similarly reflected on encounters where patients had expressed feeling lonely, but where the approach did not resonate with their emotional state or personal circumstances:

/…/I met someone who was lonely, but who didn’t want to be in a group. Then it gets a bit tricky. They were looking for a friend./…/I think there were one or two, who refused to participate, because it [the approach] wasn’t what they were looking for. But they said that they felt… felt bad because they were too lonely. (prescriber, Centre A)

In other cases, the idea of participating in social activities appeared relevant and aligned with patients’ needs, but implementation was hampered by a lack of suitable options, accessible alternatives, or other structural barriers in the outer setting (Damschroder *et al*. 2022). Some patients had specific interests or felt they had explored the available offerings, thereby expecting prescribers to identify or provide something new that they had not already discovered. This placed considerable pressure on prescribers, making it difficult to tailor the prescription meaningfully. Others lived in municipalities where the local supply of activities was limited. The absence of relevant, inclusive, and feasible options, particularly in rural areas, was widely discussed, often framed as a barrier to fulfilling the interventions’ purpose and promise.

In cases where local activities were available, implementation could also be constrained by different factors. One barrier was prescribers’ limited knowledge of available options and a perceived lack of referral pathways or collaborations linking primary care with community resources—connections that generally appeared weak and underdeveloped. As such, many prescribers described struggling to stay informed about local offerings and felt uncertain about the availability of activities. Another barrier was the logistical and psychological challenges faced by patients, such as financial restrictions, transportation difficulties, mobility limitations, or anxiety about attending unfamiliar settings. These factors made it difficult for some to engage, even when activities were available and aligned with their interests. In such instances, participation often required extended or other forms of support that were not a core part of the model, making the intervention feel like a missed opportunity. However, in a few cases, prescribers bridged this gap by helping patients navigate practical obstacles or by directly supporting transitions from prescription to participation in an activity, thus adapting the intervention to ensure that it could be meaningfully enacted.

Regardless of whether the issue was a limited supply of activities, insufficient knowledge of available options, or logistical barriers, the mismatch between patient needs or preferences and what could be offered often led to disappointment and frustration. Many prescribers shared concerns about raising expectations they could not meet. Occasionally, this seemed to have prevented them from asking about loneliness and offering the approach out of fear that they might not be able to follow through, potentially making the situation worse for the patient by surfacing unmet needs without being able to address them. Some patients also described feeling promised something that could not be delivered. For a few that participated in a prescribed activity, their experience turned out to be negative, often because it was not properly tailored to their needs. In addition, examples of distress arose when the model was not followed as intended, for instance, when patients were promised a follow-up and got their hopes up for change, only to be left waiting. As one patient from Centre I shared:

In the beginning I had high expectations because I wasn't feeling well. And then I thought, now I’ve got this going and, like, this is going to be good. Yes, and we had good conversations and there were thought-provoking questions [in the assessment]/…/So, it was all good, I thought it was good./…/But, she [the prescriber] was supposed to call me on July 11th [2024]. That’s right, and she hasn’t called yet [date of interview March 2025]. And that broke me. I was sad. That’s how it is. There’s not so much more to say.

To the extent that the Swedish social prescribing model was implemented, its enactment seems to have often contributed to feelings of disappointment, frustration, and, in some cases, even distress, thus undermining rather than fulfilling its intended purpose of alleviating loneliness and improving health among older adults. Together, these findings highlight a critical tension in the model’s operationalization: while social activities were intended to offer a personalized and empowering response to loneliness, their delivery is contingent on how the model itself is enacted, and also on its fit with patients’ needs, preferences, and expectations, as well as with the broader community context (May and Finch 2009, Damschroder *et al*. 2022).

## Concluding remarks

This process evaluation unpacked the early implementation of a social prescribing model in Sweden, aiming to assess how and under what conditions it was implemented in the ‘real-world’ context of primary care. Integrating two complementary frameworks (Schroeder *et al*. 2022) while triangulating qualitative data collected over time, the findings reveal that implementation challenges were not only about prescribers’ engagement (as highlighted by NPT) but also about structural and organizational barriers (as highlighted by CFIR). Specifically, although the model was perceived as relevant and timely, early enthusiasm waned, and implementation was constrained by several factors. These included prescribers varying perceptions of loneliness, which resulted in a selective rather than routinized use of the model’s core question. While well intentioned, such adaptations reflect a lack of fidelity to a central aspect of the intervention, which hindered implementation. Rather than being provided based on need, in line with Swedish healthcare (SFS 2017:30) policy according to patients’ subjective experiences of loneliness, social prescribing became a targeted approach, delivered according to prescribers’ discretionary judgements about who is lonely, what loneliness looks like, and when it is appropriate or not to ask about it.

Consistent with research on the implementation of social prescribing in other contexts (Chng *et al*. 2021, Calderón-Larrañaga *et al*. 2024), the results pointed to a lack of embedding within organizational workflows, constrained in our case by leadership engagement and managerial support that appeared symbolic rather than championing. This hampered implementation by positioning the intervention as peripheral and optional in relation to clinical, statutory, or more urgent tasks, limiting the model’s integration into everyday practice. Furthermore, the model’s operationalization often failed to meet older adult patients’ needs and expectations, highlighting a lack of fidelity to the person-centred process, considered key not only in the Swedish model but also in previous research (Grover *et al*. 2023). Prescriptions were also often guided more by perceived local availability of social activities than by what was meaningful to the individual. While this was sometimes due to a lack of options, particularly in rural areas, it also emerged from prescribers’ limited understanding of or familiarity with activities, particularly in the context of time constraints, insufficient collaborative structures, and other systemic barriers. However, regardless of the underlying reasons, the results suggested that the lack of person-centredness may have reinforced rather than redressed feelings of loneliness within an already marginalized group of older adults.

In sum, while the Swedish social prescribing model holds some promise, its feasibility and potential for scale-up will depend on strengthening adherence to its core principles while ensuring that both the ‘inner’ primary care setting and the ‘outer’ community context have the capacity to accommodate the intervention in line with previous research (Bertotti *et al*. 2018, Holding *et al*. 2020, Pescheny *et al*. 2020). Through a continued process of adapting the approach and developing targeted implementation strategies, this will require clearer guidance for high-fidelity delivery, stronger organizational support, and improved structures that connect primary care with local community resources. Together, these refinements could allow social prescribing to become a meaningful and sustainable part of Swedish primary care, consistent with national health system reforms (SOU 2018:39) and concerns about loneliness as a pressing problem (Swedish Public Health Agency 2025). In this light, future studies should explore how contextual variations, in Sweden and internationally, shape implementation processes and outcomes while examining strategies for tailoring the model to fit diverse settings.

## Data Availability

The data cannot be shared publicly to protect the privacy and confidentiality of participants in the study. Data will be shared on reasonable request to the corresponding author.
